# ChatGPT as a prospective undergraduate and medical school student

**DOI:** 10.1371/journal.pone.0308157

**Published:** 2024-10-23

**Authors:** Marco Giunti, Fabrizia Giulia Garavaglia, Roberto Giuntini, Giuseppe Sergioli, Simone Pinna

**Affiliations:** University of Cagliari, Cagliari, Italy; UCSI University Kuala Lumpur Campus: UCSI University, IRAN, ISLAMIC REPUBLIC OF

## Abstract

This article reports the results of an experiment conducted with ChatGPT to see how its performance compares to human performance on tests that require specific knowledge and skills, such as university admission tests. We chose a general undergraduate admission test and two tests for admission to biomedical programs: the Scholastic Assessment Test (SAT), the Cambridge BioMedical Admission Test (BMAT), and the Italian Medical School Admission Test (IMSAT). In particular, we looked closely at the difference in performance between ChatGPT-4 and its predecessor, ChatGPT-3.5, to assess its evolution. The performance of ChatGPT-4 showed a significant improvement over ChatGPT-3.5 and, compared to real students, was on average within the top 10% in the SAT test, while the score in the IMSAT test granted admission to the two highest ranked Italian medical schools. In addition to the performance analysis, we provide a qualitative analysis of incorrect answers and a classification of three different types of logical and computational errors made by ChatGPT-4, which reveal important weaknesses of the model. This provides insight into the skills needed to use these models effectively despite their weaknesses, and also suggests possible applications of our analysis in the field of education.

## 1 Introduction

The foreseeable widespread diffusion of large language models such as ChatGPT has sparked a broad debate about their potential use in many different contexts, as they could assist (or even replace, revolutionizing the labor market) workers in professions generally classified as intellectual, such as teachers, writers, journalists, programmers, and advertisers, to name a few [1].

Focusing on ChatGPT, we thought that an interesting contribution to this debate would be to ask how its performance compares to human performance on tests that require specific knowledge and abilities, such as university assessment tests. In addition, we hypothesized that these experiments would provide useful data for identifying the strengths and weaknesses of this model at the current state of the technology. This identification is, in our view, a necessary step to better specify the possible professional uses of such technologies, and also to advance some specific areas such as prompt engineering and the educational use of AI.

Evaluating ChatGPT’s performance on university tests is a common strategy, also used by its developers [2]. The novelty of our work with respect to other similar studies [3, 4] is that, in addition to the performance analysis, we provide a qualitative analysis of incorrect answers and a classification of common errors.

In Section 2 we provide some background on ChatGPT’s architecture, training, and functioning. As said, we found it interesting to compare the performance of this model to human performance on university admission tests. For this purpose, we chose three well-known admission tests. The rationale for these choices and the tests themselves are described in detail in Sections 3 and 4.

We started our testing with ChatGPT-3.5, which was the only model available at that time. At the end of this first series of tests, ChatGPT-4 was released. In order to provide a more up-to-date analysis, we decided to evaluate ChatGPT-4’s performance on the same tests that we had already administered to ChatGPT-3.5. The description of the methodology and the overall results of our testing are explained in Sections 4–6.

Although the overall performance of both models was quite good, in the course of our experiments we observed the recurrence of some errors, especially logical and computational ones. In our view this phenomenon was indicative of typical weaknesses in the model and thus deserved closer attention. We then identified situations in which the models made these recurrent errors, isolating macro problem areas that reveal some specific and important deficiencies in the models. This also provides some insight into the skills needed to use these models effectively despite their weaknesses. In Section 7.1 we discuss the overall performance of the models. In Section 7.2 we then provide a classification and a qualitative analysis of the recurrent errors, as well as some typical examples, and we then discuss possible applications of our analysis in the field of education.

## 2 What is ChatGPT and how does it work?

ChatGPT (Generative Pre-trained Transformer) is a cutting-edge language model developed by OpenAI that leverages deep learning techniques to generate contextually relevant and coherent text in response to user input.

Once trained, ChatGPT can generate text in response to user input by leveraging its learned knowledge of language. When a user enters a prompt or a question, ChatGPT processes the input and generates a response based on its understanding of the context and the language. The output generated by ChatGPT is often coherent and contextually relevant, making it a valuable tool for a wide range of applications, including customer service, language translation, and content creation.

One of the key strengths of ChatGPT is its ability to generate text that is both fluent and diverse. Unlike traditional rule-based systems that generate text based on a predefined set of rules [5], ChatGPT is capable of generating text that is free-form and can adapt to different writing styles and genres.

But, from a technical viewpoint, how does the ChatGPT training algorithm work? ChatGPT uses an *unsupervised learning* algorithm [6] to train its neural network. Unsupervised learning is a type of machine learning in which the model is presented with unlabeled data and must find patterns and relationships within the data on its own. The specific algorithm used to train ChatGPT is called the *transformer architecture* that was first introduced in 2017 [7] and has since become the state-of-the-art for many natural language processing tasks.

The transformer architecture consists of a series of encoder and decoder layers. Each layer consists of multiple attention heads, which allow the model to focus on different parts of the input data. The attention mechanism is a key innovation of the transformer architecture and allows the model to learn contextual relationships between words in a sentence.

In terms of model size, [8] reports that GPT-3, a model that GPT-3.5 improves upon, contains 175 billion trainable parameters. In the same paper, the authors compare the performance of the model when the number of parameters is set to different orders of magnitude, and show how the size of the model is directly related to performance. For example, three different models were tested in a simple task that required the model to remove random symbols from a word in a given a number of example in context. The smaller model, with 1.3 billion parameters, reached about 5% of accuracy; a model with 13 billions parameter reached about 25% of accuracy; finally GPT3, with 175 billion parameters, reached about 65% of accuracy in the task. [8, p. 4]. Although the model is not directly inspired by brain function, it is interesting to note that the number of trainable parameters of the model is comparable to the number of neurons in the human brain (about 100 billion), while the number of brain synapses (between 10^14^ and 10^15^) is several orders of magnitude larger than that of the model [9, Section 7.3].

During training, ChatGPT is presented with a large corpus of text, such as the entire text of Wikipedia. The model is then trained to predict the next word in a sequence of words. For example, given the input sequence “The cat is on the”, the model is trained to predict the next word “table” or “chair” or any other appropriate word based on the context.

To improve performance, the training process is typically done using a technique called *batch training* [10]. Batch training involves presenting the model with multiple examples at once and updating the model weights based on the average loss over the entire batch. This helps the model to learn from a diverse set of examples and improves its ability to generalize to new inputs.

Moreover, the training process is typically done using *stochastic gradient descent* (SGD) [11] or a variant thereof. SGD involves updating the weights of the neural network in the direction of the steepest descent of the loss function with respect to the weights. This process is repeated many times, with the model updating its weights after each batch of examples.

Another method used by ChatGPT during the training is the so called *reinforcement learning* (RL) [12]. Reinforcement learning is a type of machine learning that enables an AI model to learn from its environment through trial and error [13]. In the case of ChatGPT, the model is trained on a large dataset of text-based interactions, and it uses RL to continuously improve its responses based on user feedback. When a user interacts with ChatGPT, the model generates a response, and the user provides feedback on whether the response was helpful or not. This feedback is then used to update the model, so that it can improve its future responses. By using RL, ChatGPT can adapt to changing user needs and preferences over time, and improve its performance in real-world applications. Below, we highlight some aspects of learning processes that are useful for an introduction to this type of model. However, we cannot delve into the general aspects of RL and Deep Learning because they are beyond the scope of this research (more technical details on the architecture and training processes of GPT3, upon which ChatGPT-3.5 is based, are in [8]).

It’s worth noting that ChatGPT uses many techniques together to learn from data and generate responses. The model also relies on standard machine learning techniques, such as deep learning and natural language processing, to understand and generate human-like responses.

After training, the ChatGPT algorithm generates responses to user input using a process called *inference*. Inference involves passing the user input through the pre-trained neural network to generate a response.

To generate an optimal response, the algorithm first processes the user input by tokenizing it into a sequence of integers that represent the words in the input. The algorithm then passes the tokenized input through the pre-trained neural network. During inference, the algorithm uses a technique called *beam search* [14] to generate the response. Beam search is a search algorithm that generates a sequence of tokens by iteratively selecting the most probable token at each step. The algorithm generates a set of possible responses and selects the one with the highest probability score. The probability score for each response is calculated by the neural network using a *softmax function* [15]. The softmax function normalizes the output of the neural network into a probability distribution over the possible words in the response. The algorithm then selects the highest probability word as the next token in the response.

To ensure coherence and relevance of the response, the algorithm also uses a technique called *top-k sampling* [16]. Top-k sampling restricts the algorithm to only consider the top-k most probable words at each step, where the parameter k refers to the number of most probable words chosen for optimizing the process. For example, if k = 5, then only the most probable 5 words will be used at each step of the algorithm. This ensures that the response is relevant to the input and avoids generating nonsensical responses.

Once the algorithm generates a complete response, it returns the response to the user. The response is typically post-processed to remove any special tokens or formatting and is presented to the user as plain text.

Overall, the ChatGPT algorithm generates responses to user input using a combination of pre-trained neural networks, beam search, and top-k sampling. This allows the algorithm to generate coherent and contextually relevant responses that are tailored to the user’s input.

In March 2023, OpenAI developed GPT-4, a new multimodal model capable of accepting both image (visual version) and text inputs (non-visual version) and producing text outputs. Currently, only the non-visual version is available to general users. GPT-4 represents a natural evolution of its predecessor, GPT-3.5. OpenAI has provided an extensive comparison between GPT-3.5 and GPT-4, which can be found on their website (https://openai.com/research/gpt-4). According to the developers, GPT-4 exhibits more reliable and creative performance compared to GPT-3.5 and appears to handle more nuanced instructions. This has been demonstrated through various benchmarks and tests comparing the two versions.

To achieve this new version, OpenAI rebuilt its entire deep learning stack and co-designed a supercomputer from the ground up with Azure for its workload, resulting in a highly stable training run for GPT-4. The model’s text input capability is available through ChatGPT and the API, while image input capability is currently being prepared for wider availability. GPT-4 has been used internally to demonstrate strong performance in support, sales, content moderation, programming, and AI output evaluation. However, developers still acknowledge some limitations, particularly in making reasoning errors. OpenAI describes GPT-4 as an ongoing system, with further developments to be provided soon; [2] is a detailed technical report on GPT-4’s new features and differences from GPT-3.5. It is worth noting that, as demonstrated in the experimental sections of the paper, although GPT-4’s average performance generally surpasses GPT-3.5, it is not uncommon for some questions to be answered correctly by GPT-3.5 but not by GPT-4.

## 3 The question

The goal of our work was twofold. First, to evaluate the performance of ChatGPT on problems designed to test cognitive requirements for university access and to compare it with the performance of real students. Second, to focus on cases where the model fails and to identify classes of errors that highlight particular shortcomings or limitations of ChatGPT.

More specifically, we wondered how the two versions of ChatGPT would perform on tests that are usually required for admission to a university program. We think this is an interesting question for at least three reasons. First, it allows us to obtain objective data about the performance of the two systems that can be compared to the performance of students who took the same test. Second, it can also suggest new questions about the structure and design of admissions tests themselves and, more generally, about what knowledge and skills it is most appropriate to require of a student preparing to enter college today. Third, this comparison can give us insight into the overall ability of the two systems to handle this type of test, as well as their weaknesses or, conversely, strengths in answering specific types of questions.

Since the model’s answers are often articulated and make explicit the procedures it uses to arrive at a given solution, it is indeed interesting to assess the level of accuracy of these procedures and the degree of confidence the model attributes to them. The analysis of incorrect answers reveals recurrent errors that do not necessarily depend on the level of difficulty of the questions posed. This aspect can be explored through a systematic analysis of the error cases to try to identify specific classes that capture similar errors on different problems. In this way, we have identified three distinct error classes. Each of them includes several error cases of the same type and highlights a system weakness that deserves special attention.

Among the many admission tests that could be studied, we chose the Scholastic Assessment Test (SAT), a standardized test used primarily in the United States as a criterion for college admission, the Cambridge BioMedical Admission Test (BMAT), a test used by many universities around the world for admission to biomedical programs, and the Italian Medical School Admission Test (IMSAT), the test required for admission to any medical school in Italy.

These tests are structured to assess the possession of general logic and problem-solving skills, as well as domain-specific knowledge, which are necessary to effectively solve the problems presented. By evaluating ChatGPT’s performance on these tests, it is therefore possible to assess different types of skills, both specific and cross-disciplinary.

### 3.1 The Scholastic Assessment Test (SAT)

The SAT (Scholastic Assessment Test) is a standardized test used primarily in the United States as a criterion for college admissions. It assesses a candidate’s proficiency in reading, writing, and mathematics and provides an overall score out of 1600.

The SAT was introduced in 1926 and is developed and administered by the College Board, a non-profit organization dedicated to college education. The original name of the test was “Scholastic Aptitude Test”, but it was changed to “Scholastic Assessment Test” in 1993 to reflect the idea that the test evaluates acquired skills during high school, rather than innate aptitude.

The SAT is typically taken by high school students during their junior or senior year, and the scores are used by colleges and universities as a factor in their admission decisions. The test is composed of two sections: Evidenced-based Reading and Writing, and Math.

The Evidence-based Reading and Writing section is made up of a Reading Test and a Writing and Language Test, each composed of multiparagraph passages and multiple-choice questions. The Reading Test measures comprehension and reasoning skills and focuses on close reading of passages in a wide array of subject areas. The Writing and Language Test measures a range of skills, including command of evidence, expression of ideas, and the use of standard English conventions in grammar and punctuation.

The Math section includes multiple-choice and student produced response questions, and it is divided into two parts: one that allows calculator use and one that does not. It assesses skills in algebra, problem solving and data analysis, manipulation of complex equations, geometry, and trigonometry.

Students receive a total score that is the sum of their scores on the two sections. To calculate section scores, the student’s raw score—the number of questions correctly answered—for each section is calculated first. Nothing is deducted for incorrect answers or for unanswered questions. Next, each of the raw section scores is converted to a scaled score of 200–800. This conversion process adjusts for slight differences in difficulty among versions of the test and provides a score that is consistent across different versions. The scaled scores are the scores provided on score reports. On the paper-based test, students receive additional scores beyond the total and section scores, namely, cross-test scores and subscores.

The range for cross-test scores is 10–40. These scores represent performance on selected questions across the three tests in the two sections and show a student’s strengths in the following domains: Analysis in History/Social Studies; Analysis in Science.

Subscores, ranging from 1 to 15, provide feedback on student performance in seven skill areas: Command of Evidence, Words in Context, Expression of Ideas, Standard English Conventions, Heart of Algebra, Problem Solving and Data Analysis, Passport to Advanced Math. The first four subscores represent performance on selected questions from the two tests in the first section; the last three represent performance on selected questions from the math test in the third section.

### 3.2 The Cambridge BioMedical Admission Test (BMAT)

The BMAT is used by universities around the world to select applicants for medical, dental, biomedical and veterinary programs. It tests the ability to apply scientific and mathematical knowledge, as well as problem solving, critical thinking, and written communication skills that are essential for study at the university level. The test scores are intended to be used as one component of the selection decision in conjunction with other information available to admissions tutors.

Test items draw upon general academic skills and basic science knowledge, rather than recent specialist teaching. The test provides an objective basis for comparing candidates from different backgrounds, including mature applicants and those from different countries.

The test is divided into three sections. The first two sections are in multiple-choice format and there are no penalties for incorrect answers, so candidates should attempt all questions. The third section requires candidates to write a short essay.

For each of the first two sections, the raw score is the number of correct answers. The corresponding BMAT scores, on a scale from 1 to 9, are obtained from raw scores by means of a conversion table provided by Cambridge Assessment Admissions Testing. The conversion adjusts for differences in difficulty among versions of the test and yields a score that is consistent across different versions.

The third section is scored by Cambridge Assessment Admissions Testing examiners. Scores are reported for the quality of content on a scale from 1 to 5, and for the quality of English on a scale from A to E. An image of the response is also supplied to each institution to which the candidate has applied. This, in addition to the scores of the third section, provides the institution with a basis for qualitative assessments of writing skills.

The name, structure and content of the first section of the test have changed since 2020. The number of questions in each subject area in the second section has also changed from 2020. The three sections, and their relevant changes since 2020, are outlined below.

1. Before 2020, Section 2 was called *Aptitude and Skills* and tested generic skills in three broad areas: Problem solving (13 questions), Understanding argument (10 questions), Data analysis and inference (12 questions).
  Since 2020, Section 2 has been renamed *Thinking Skills* and there are just two areas called “Critical thinking” and “Problem solving”. The questions in the first area correspond to those in the “Understanding argument” area of the previous years, but those in the “Problem solving” area also include some questions that would have previously belonged to the “Data analysis and inference” area. Also the number of questions has changed, as there are 16 questions in multiple-choice format for each of the two areas.
  Problem solving requires candidates to solve problems, using simple numerical operations; it requires the capacity to select relevant information, identify similarity, determine and apply appropriate procedures.
  Critical Thinking presents a series of logical arguments and requires respondents to summarize conclusions, draw conclusions, identify assumptions, assess the impact of additional evidence, detect reasoning errors, match arguments, apply principles.
2. Section 3, *Scientific Knowledge and Applications*, tests whether candidates have an appropriate level of scientific knowledge and the ability to apply it. Questions are limited to material typically found in general science and mathematics courses at the secondary school level.
  Section 3 has a total of 27 questions and calculators are not allowed. Before 2020, there were 6–8 questions each for biology, chemistry and physics, and 5–7 questions for mathematics. Since 2020, there have been 7 questions each for biology, chemistry and physics, and 6 questions for mathematics.
3. Section 4, *Writing Task*, offers the candidate a choice of three writing assignments from which to choose one. Answers are strictly limited to one A4 page.

### 3.3 The Italian Medical School Admission Test (IMSAT)

The IMSAT is the test used in Italy to gain admission to any medical school in the country. The test is administered on a national basis by the Ministry of University and Research. It consists of 60 multiple-choice questions, with 5 possible answers and only one correct answer. A correct answer is worth 1.5 points, while an incorrect answer is worth -0.4 points. The candidate may choose not to answer, and each unanswered question is worth 0 points. A score of 20 or higher is required to pass the test.

If the candidate passes the test and obtains a score equal to or higher than a cutoff score, he/she is allowed to enroll in one of the Italian medical schools, according to a national ranking that depends on the number of places available and the number of candidates who took the test. The test is held once a year, usually in the first week of September, and admissions for the academic year in question are based on the results.

For example, the 2022 test was held on 6–9-2022 and was used for admissions for the 2022–2023 academic year. According to the national ranking published on 29–9-2022, the cutoff score for admission to a medical school nationwide was 33.4. Scores at or above 33.4 allowed admission to different medical schools, depending on the cutoff score required by each institution. The school with the highest cutoff score was that of the University of Milan Bicocca (54.5); the school with the lowest cutoff score was that of the University of Messina (33.4). There were 56,775 test takers, but only 28,793 passed the test with a score of 20 or higher, for a pass rate of 50,71%. However, not all those who passed the test could be admitted, as there were only 14,740 places available (data taken from https://www.tecnicadellascuola.it/test-medicina-2022-una-strage-la-meta-dei-partecipanti-non-prende-neanche-il-punteggio-minimo and https://www.studenti.it/test-medicina-2022-posti-disponibili.html?gallery=63962).

The test is divided into several sections, each designed to test a specific area of knowledge and skills. However, the division into sections has changed over the years, as has the number of questions in each section. For this reason, the following description refers only to the 2019, 2020, and 2022 tests. These are the three tests that we administered to both ChatGPT-3.5 and ChatGPT-4 in our experiment. The reasons for this choice are explained in Section 4. It should also be noted that, for next year’s test, the Ministry of University and Research has announced a number of changes, some of them quite radical, to the structure of the test.

The 2019 and 2020 tests are divided into 4 sections. The first, called “Test of Logical Reasoning and General Knowledge”, consists of 22 questions. There are 9 questions in the Logical Reasoning area. They are designed to test the candidate’s ability to follow an argument logically or to solve simple problems. There are 13 questions in the General Knowledge area. They are designed to test the ability to understand written texts in Italian or to probe general cultural skills and knowledge acquired in previous studies. The remaining three sections, “Biology Test”, “Chemistry Test”, and “Physics and Mathematics Test”, are designed to test knowledge and skills in the respective disciplines. These skills and knowledge correspond to the preparation provided by upper secondary schools. The Biology Test consists of 18 questions, the Chemistry Test of 12 questions, and the Physics and Mathematics Test of 8 questions.

The 2022 test is divided into 5 sections. The first, called “Test of Reading Skills and Knowledge Acquired in Studies”, corresponds to the General Knowledge area of the 2019 and 2020 tests, but it consists of only 4 questions, instead of the 13 questions in the two previous tests. The second section, “Test of Logical Reasoning and Problems”, corresponds to the Logical Reasoning area of the two previous tests, but it consists of 5 questions instead of 9. The remaining three sections “Biology Test”, “Chemistry Test”, and “Physics and Mathematics Test”, consist of 23, 15 and 13 questions respectively.

## 4 Materials and methods

The reasons for selecting the previously described tests are varied. First and foremost, they provide a wide variety of questions across a range of problem categories that span several macro-areas, including logic, language production, critical thinking, mathematics, biology, and chemistry. These tests are designed to assess a wide range of skills and are the result of many years of collaboration between experts with diverse expertise in the disciplines studied, in learning processes, and in educational practice in general.

Two of the selected tests, SAT and BMAT, are among the most widely used and are considered essential for evaluating core skills and abilities that are a prerequisite for admission to a particular university program [17, 18]. Because of the widespread use of these assessment tools, a very large number of students are assessed using this type of test. This provides a very large database of scores from which to evaluate ChatGPT performance. While some of these tests set a cut score, others base the passing score on the number and quality of answers. Either way, this provides a large amount of data to evaluate the performance of ChatGPT.

The Italian IMSAT, like the BMAT and SAT, offers a large number of questions covering a variety of subject areas. Unlike these tests, the IMSAT does not have the same widespread international use, as it is administered in Italian and is only used in Italy for university admission to medical schools. However, given the large number of students interested in these disciplines, the data available are very extensive. Interestingly, unlike the other tests, it provides a cut score that varies from year to year, making it possible to compare ChatGPT performance with that of real students for any given year.

Compared to the other two tests, the SAT provides a more detailed analysis of the results. In fact, the SAT provides specific scores that are obtained by cross-referencing some of the answers to specific questions in different sections, thus allowing for cross-sectional skill assessments.

All the three tests were administered in their original languages: English for the SAT and BMAT, Italian for the IMSAT.

Initially, we used a free account to access the ChatGPT platform (chat.openai.com), through which we administered tests to ChatGPT-3.5. After the release of ChatGPT-4, we used a ChatGPT Plus account to administer questions to the newer model.

The questions were administered one at a time in the first phase of testing (on ChatGPT3.5). Later, given ChatGPT-4’s greater ability to handle long texts, we administered groups of 2–5 questions at a time.

## 5 The experiment

Our experiment consisted in having ChatGPT-3.5 and ChatGPT-4.0 take three versions of each of the three tests SAT, BMAT, IMSAT.

For the SAT, we chose the official practice tests published by College Board on their website (https://satsuite.collegeboard.org/sat/practice-preparation/practice-tests. In particular, we chose the three more recent full length practice tests for assistive technology (tests #8, #9, #10). The reason for this choice is that the assistive technology practice tests include full textual descriptions of images and graphs, so that all questions in these tests can be submitted to ChatGPT. In fact, ChatGPT-3.5 only accepts textual input, so it is not able to process questions based on images or graphs. According to OpenAI, GPT-4 no longer has this limitation, as it is a multimodal system that accepts image or text input, and produces text output. However, the ability to interpret non-textual input was not implemented in ChatGPT-4 when we conducted our experiment. The version we used was only capable of intepreting textual input.

For the BMAT, we chose three past tests published by Cambridge Assessment Admission Testing. Specifically, we chose the two published most recent past tests (2021 and 2020) and the 2018 test. We did not choose the 2019 test because it contained a higher number of questions with images or graphs than the 2018 test, namely, 16/35 in the first section and 8/27 in the second section, compared to 3/35 and 4/27.

For the IMSAT, we chose three past tests, the most recent one (2022) and the 2020 and 2019 tests. We did not choose the 2021 test because it had a higher number of questions with images or graphs than either of the previous two tests.

Each test was administered in its original language, English for the SAT and BMAT, and Italian for the IMSAT. The details of the three administration procedures are described below.

### 5.1 Administering the SAT test

The three SAT practice tests for assistive technology (#8, #9, #10) were given first to ChatGPT-3.5 and then to ChatGPT-4 according to the following procedure.

For each test, we administered all the questions in each of the two test sections. For questions with images, graphs, or tables, we used the full textual descriptions provided by the test itself.

For each test, and each version (3.5 and 4) of ChatGPT, the questions were copied from the text published on the College Board website (https://satsuite.collegeboard.org/sat/practice-preparation/practice-tests), and then pasted into ChatGPT dialogue box. For multiple choice questions, ChatGPT was asked to indicate the correct answer; ChatGPT responded to each question by selecting only one of the possible options and explaining the reasons for that choice. For math questions that required a student produced response, we simply submitted the full question; ChatGPT then generated its answer and explained the solution procedure. We saved the entire dialogue, which can be found in the S1 File of this paper.

### 5.2 Administering the BMAT test

The three BMAT tests from October 2018, November 2020, and November 2021 were given first to ChatGPT-3.5 and then to ChatGPT-4 according to the following procedure.

For the 2018 test, we administered questions from Section 1 (Aptitude and Skills) and Section 3 (Scientific Knowledge). For the 2020 and 2021 tests, we administered questions from Section 1 (Thinking Skills) and Section 2 (Scientific Knowledge). We did not administer Section 3 (Writing Task) of any of the tests because we could not get a score for this section, as the score is provided by Cambridge Assessment Admissions Testing examiners. In addition, we did not administer questions that included images or graphs because, as noted above (see Section 5), either version of ChatGPT is not able to process them. For tables, we transcribed them into Latex or provided a detailed textual description of them. Below is the number of questions we actually submitted to the two versions of ChatGPT for each of the three BMAT tests:

- BMAT 2018: 32 out of 35 questions for Section 1; 23 out of 27 questions for Section 2.
- BMAT 2020: 26 questions out of 32 for Section 1; 20 questions out of 27 for Section 2.
- BMAT 2021: 25 questions out of 32 for Section 1; 22 questions out of 27 for Section 2.

For each test, and each version (3.5 and 4) of ChatGPT, the questions were copied from the text provided by Cambridge Assessment Admissions Testing, and then pasted into the ChatGPT dialogue box, with ChatGPT being asked to indicate the correct answer. ChatGPT responded to each question by selecting only one of the possible options and explaining the reasons for that choice. We saved the full dialogue, which can be found in the S1 File of this paper.

### 5.3 Administering the IMSAT test

The three IMSAT tests of 2019, 2020 and 2022 were given first to ChatGPT-3.5 and then to ChatGPT-4. The procedure was as follows.

For each test, and each version (3.5 and 4) of ChatGPT, each question was copied from the official text published by the Ministry of University and Research and pasted into the ChatGPT dialogue box, together with a request to indicate which of the five answers was the correct one. Using this procedure, we administered all the 60 multiple choice questions of the 2019 and 2022 tests. For the 2020 test, we administered 59 questions, because one of the biology questions was based on an image that, as mentioned earlier (see Section 5), cannot be processed by either version of ChatGPT. ChatGPT answered each question by selecting only one of the five options and explaining the reason for its choice. For each test, we saved the full dialogue, which can be found in the S1 File of this paper.

Before deciding on this administration procedure, we also tried a slightly different procedure, in which we asked ChatGPT to choose one of five options or, if it was unsure of the answer, to choose none. This procedure would have been more similar to the way the test is actually administered to students, who also have the option of not answering if they are unsure. However, we found that, for a significant number of trial questions, ChatGPT always produced an answer and always chose exactly one of the five options. As there were no significant differences in the types of answers produced by the two procedures, we ultimately decided to use the simpler procedure described above.

## 6 Experiment results

This section shows and comments the results of ChatGPT-3.5 and ChatGPT-4 on the three versions of the tests SAT, BMAT, IMSAT.

### 6.1 SAT

Table 1 shows the results of ChatGPT-3.5 and ChatGPT-4 in the three tests SAT#8, SAT#9 and SAT#10. Raw scores are provided for all parts (Reading Test; Writing and Language Test; Math Test—No Calculator; Math Test—Calculator) of the two test sections. The scores of the two test sections (*Reading and Writing*; *Math*), and the total scores are also provided.

**Table 1:** SAT results. The table shows the results obtained by ChatGPT-3.5 (above) and ChatGPT-4 (below) in the three tests SAT#8, SAT#9, SAT#10. For each part of the two test sections, the raw score is expressed in terms of correct answers/questions administered, and it is followed by the corresponding percentage of correct answers. The raw score for the two sections and the total raw score, with the corresponding percentages, are also shown. The score for each section and the total score, followed by the corresponding percentiles, are shown in bold.

	**SAT#8 ChatGPT-3.5**	**SAT#9 ChatGPT-3.5**	**SAT#10 ChatGPT-3.5**
**Reading**	36/52 69%	43/52 82%	42/52 81%
**Writing**	33/44 75%	30/44 68%	34/44 77%
**Subtotal (Reading and Writing)**	69/96 72%	73/96 76%	76/96 79%
**Math no calc.**	13/20 65%	13/20 65%	15/20 75%
**Math calc.**	18/38 47%	28/38 73%	26/38 68%
**Subtotal (Math)**	31/58 53%	41/58 71%	41/58 71%
**Total**	100/154 65%	114/154 74%	117/154 76%
**Score (Reading and Writing)**	**600 (73rd perc.)**	**630 (81st perc.)**	**610 (76th perc.)**
**Score (Math)**	**560 (64th perc.)**	**630 (81st perc.)**	**600 (75th perc.)**
**Score (Total)**	**1160 (69th perc.)**	**1260 (82nd perc.)**	**1210 (76th perc.)**
	**SAT#8 ChatGPT-4**	**SAT#9 ChatGPT-4**	**SAT#10 ChatGPT-4**
**Reading**	48/52 92%	50/52 96%	50/52 96%
**Writing**	37/44 84%	39/44 88%	39/44 88%
**Subtotal (Reading and Writing)**	85/96 89%	89/96 93%	89/96 93%
**Math no calc.**	16/20 80%	16/20 80%	16/20 80%
**Math calc.**	28/38 73%	30/38 79%	28/38 73%
**Subtotal (Math)**	44/58 76%	46/58 79%	44/58 76%
**Total**	129/154 84%	135/154 88%	133/154 86%
**Score (Reading and Writing)**	**690 (92nd perc.)**	**730 (97th perc.)**	**690 (92nd perc.)**
**Score (Math)**	**680 (89th perc.)**	**680 (91st perc.)**	**620 (79th perc.)**
**Score (Total)**	**1370 (91st perc.)**	**1410 (87th perc.)**	**1410 (87th perc.)**

Table 2 shows some aggregate percentages (i.e., the percentages of correct answers relative to the total number of questions submitted in each part of the three tests), the average section scores, and the average total score.

**Table 2:** SAT results. Aggregate percentages and average scores for SAT#8, SAT#9, SAT#10. The table shows, for each part of the two test sections, the percentage of correct answers relative to the total number of questions submitted in the three tests. In addition, for each score, the average score in the three tests is given.

SAT#8, SAT#9, SAT#10	Aggregate ChatGPT-3.5	Aggregate ChatGPT-4
**Reading**	78%	95%
**Writing**	73%	87%
**Subtotal (Reading and Writing)**	76%	91%
**Math no calc.**	68%	80%
**Math calc.**	63%	75%
**Subtotal (Math)**	65%	77%
**Total**	72%	86%
**Score (Reading and Writing)**	**610 (76th perc.)**	**700 (93rd perc.)**
**Score (Math)**	**600 (75th perc.)**	**660 (86th perc.)**
**Score (Total)**	**1210 (76th perc.)**	**1360 (90th perc.)**

Table 3 shows the SAT subscores for the seven skill areas (see Section 3.1) obtained by both models in each of the three tests, while Table 4 shows the aggregate percentages of correct answers for each skill area.

**Table 3:** SAT subscores results. The table reports the performance of ChatGPT-3.5 (above) and ChatGPT-4 (below) in the seven skill areas of each test. The subscore in each skill area, on a scale from 1 to 15 (bold) is followed by the raw score (correct answers/questions administered) and by the corresponding percentage of correct answers.

	**SAT#8 ChatGPT-3.5**	**SAT#9 ChatGPT-3.5**	**SAT#10 ChatGPT-3.5**
**Subscore**	**1–15** (raw)	**1–15** (raw)	**1–15** (raw)
**Command of evidence**	**13** (16/18) 89%	**8** (8/18) 44%	**9** (11/18) 61%
**Words in context**	**13** (16/18) 89%	**13** (16/18) 89%	**10** (15/18) 83%
**Standard English conventions**	**13** (18/20) 90%	**10** (15/20) 75%	**13** (19/20) 95%
**Expression of ideas**	**10** (15/24) 62%	**10** (15/24) 62%	**9** (15/24) 62%
**Passport to advanced math**	**10** (8/16) 57%	**13** (12/16) 75%	**14** (14/16) 87%
**Problem solving and data analysis**	**9** (8/17) 47%	**12** (13/17) 76%	**11** (13/17) 76%
**Heart of algebra**	**12** (16/19) 84%	**10** (14/19) 74%	**9** (12/19) 63%
	**SAT#8 ChatGPT-4**	**SAT#9 ChatGPT-4**	**SAT#10 ChatGPT-4**
**Subscore**	**1–15** (raw)	**1–15** (raw)	**1–15** (raw)
**Command of evidence**	**14** (17/18) 94%	**15** (17/18) 94%	**13** (16/18) 89%
**Words in context**	**13** (16/18) 89%	**14** (17/18) 94%	**18** (18/18) 100%
**Standard English conventions**	**14** (19/20) 95%	**14** (19/20) 90%	**12** (18/20) 90%
**Expression of ideas**	**11** (18/24) 75%	**14** (21/24) 87%	**12** (18/24) 87%
**Passport to advanced math**	**11** (10/16) 71%	**13** (13/16) 81%	**13** (13/16) 81%
**Problem solving and data analysis**	**13** (13/17) 76%	**15** (15/17) 88%	**13** (15/17) 88%
**Heart of algebra**	**12** (16/19) 84%	**10** (14/19) 74%	**10** (14/19) 74%

**Table 4:** SAT subscores results. Aggregate percentages for SAT#8, SAT#9, SAT#10. For each skill area, the table shows the percentage of correct answers relative to the total number of questions submitted in the three tests.

SAT#8, SAT#9, SAT#10	Aggregate ChatGPT-3.5	Aggregate ChatGPT-4
**Command of evidence**	65%	93%
**Words in context**	87%	94%
**Standard English conventions**	87%	92%
**Expression of ideas**	62%	83%
**Passport to advanced math**	71%	75%
**Problem solving and data analysis**	66%	84%
**Heart of algebra**	74%	77%

Finally, Table 5 shows the results obtained in both SAT cross-tests (Analysis in History and Social Sciences, Analysis in Science) for each of the three tests, and also the aggregate percentages of correct answers for both SAT cross-tests.

**Table 5:** SAT cross-tests results. The upper part of the table shows the performance of ChatGPT-3.5 and ChatGPT-4 in both cross-tests for each of the three tests. The score obtained in each cross-test, ranging from 10 to 40, is shown in bold; it is followed by the raw score (correct answer/administered questions) and by the corresponding percentage of correct answers. The lower part of the table shows the cross-tests aggregate percentages for SAT#8, SAT#9, SAT#10, that is to say, for each cross-test, the percentage of correct answers relative to the total number of questions submitted in the three tests.

	**SAT#8 ChatGPT-3.5**	**SAT#9 ChatGPT-3.5**	**SAT#10 ChatGPT-3.5**
**Cross-test**	**score 10–40** (raw)	**score 10–40** (raw)	**score 10–40** (raw)
**Analysis in History and Social Sciences**	**29** (24/35) 68%	**34** (29/35) 83%	**30** (26/35) 74%
**Analysis in Science**	**31** (24/35) 68%	**35** (29/35) 83%	**31** (26/35) 74%
	**SAT#8 ChatGPT-4**	**SAT#9 ChatGPT-4**	**SAT#10 ChatGPT-4**
**Cross-test**	**score 10–40** (raw)	**score 10–40** (raw)	**score 10–40** (raw)
**Analysis in History and Social Sciences**	**33** (29/35) 83%	**37** (32/35) 91%	**34** (31/35) 88%
**Analysis in Science**	**36** (31/35) 88%	**39** (34/35) 97%	**36** (33/35) 94%

**Aggregate SAT#8, SAT#9, SAT#10**		
**Cross-test**	**ChatGPT-3.5**	**ChatGPT-4**
**Analysis in History and Social Sciences**	75%	88%
**Analysis in Science**	75%	93%

Table 1 also shows that ChatGPT-3.5 scores are in quite good percentiles (we refer to the percentiles of 2022 SAT takers reported by College Board at https://satsuite.collegeboard.org/media/pdf/understanding-sat-scores.pdf). In test #8, its total score is in the 69^*th*^ percentile (73^*rd*^ in the *Reading and Writing* section, 64^*th*^ in the *Math* section); in test #9, it is in the 82^*nd*^ percentile (81^*st*^ in the *Reading and Writing* section, 81^*st*^ in the *Math* section); in test #10, it is in the 76^*th*^ percentile (76^*th*^ in the *Reading and Writing* section, 75^*th*^ in the *Math* section). However, ChatGPT-4 does significantly better: in test #8, its total score is in the 91^*st*^ percentile (92^*nd*^ in the *Reading and Writing* section, 89^*th*^ in the *Math* section); in test #9, it is in the 94^*th*^ percentile (97^*th*^ in the *Reading and Writing* section, 91^*st*^ in the *Math* section); in test #10, it is in the 87^*th*^ percentile (92^*nd*^ in the *Reading and Writing* section, 79^*th*^ in the *Math* section).

The data in Table 1 show a significant improvement of ChatGPT-4 over ChatGPT-3. However, both versions of ChatGPT perform better in the *Reading and Writing* section than in the *Math* section, as can be seen from the aggregate percentages reported in Table 2.

The improvements of ChatGPT-4 are basically confirmed by Table 3, which shows the subscores in all seven skill areas of the test. The aggregate percentage of correct answers for each skill area is shown in Table 4. It is worth noting that the two skill areas where ChatGPT-4 does not reach 80% correct (i.e., Passport to advanced math and Heart of algebra) are also those where it shows small improvements (4% and 3%, respectively) over ChatGPT-3.5.

The results of the SAT cross-tests (Table 5) basically confirm the above considerations. Both cross-tests (Analysis in History and Social Sciences, Analysis in Science) contain questions that focus strongly on text comprehension and data analysis, where both versions of ChatGPT show high performance. As the two cross-tests contain similar types of questions, there are no significant differences in performance between the two. In each case, the aggregate score obtained by ChatGPT-3.5 is the same, while the aggregate scores obtained by ChatGPT-4 differ by only 5%. Nevertheless, these results also show a significant improvement of ChatGPT-4 over ChatGPT-3.5.

### 6.2 BMAT

Tables 6–8 show the results of administering the three BMAT tests to the two versions of ChatGPT. Given the significant number of questions in these tests that could not be administered (see Section 6.2), we decided to normalize the raw scores obtained in each test section over the total number of questions expected from the standard BMAT format. We then converted the normalized raw scores to 1–9 scaled scores by means of the conversion tables provided by Cambridge Assessment Admissions Testing. This allows ChatGPT scores to be compared with the average scores of UCL (University College London) applicants, interviewed applicants, and offer holders (data drawn from https://www.uniadmissions.co.uk/bmat/guides/ucl-bmat-cutoff/ and https://www.theukcatpeople.co.uk/medical-schools/bmat/how-universities-use-the-bmat). For each test score, the corresponding percentile (relative to all test takers for each year) is also displayed. Percentiles refer to the scores of all BMAT test takers for each year and they are derived from the official data on the BMAT results published each year by Cambridge Assessment Admissions Testing. The three documents for the years 2018, 2020, 2021 can be found at: https://www.ox.ac.uk/sites/files/oxford/media_wysiwyg/516122-bmat-explanation-of-results-2018.pdf; https://www.ox.ac.uk/sites/files/oxford/media_wysiwyg/104532-bmat-explanation-of-results-2012.pdf; https://www.blackstonetutors.com/bmat-updates-2022-overview-of-2021-scores/.

**Table 6:** BMAT results (October 2018). The table shows the results obtained by ChatGPT-3.5 (left) and ChatGPT-4 (right) in the October 2018 BMAT test. For Sections 1 and 2 of the test, we report the number of questions administered, the raw scores (correct answers/questions administered) in each subject area and in the whole section, the normalized raw score, and the corresponding BMAT score followed by the relative percentile. At the bottom of each section, the UCL applicants, interviewed, and offer holders average scores are reported. The bottom row shows the ratio of the total number of correct answers to the total number of questions administered and the corresponding percentage.

**Section 1: Aptitude and skills**		
	**BMAT Oct. 2018 ChatGPT-3.5**	**BMAT Oct. 2018 ChatGPT-4**
**administered**	32/35	32/35
**Understanding argument**	6/10 60%	8/10 80%
**Problem solving**	0/10 0%	1/10 10%
**Data analysis and inference**	2/12 17%	7/12 58%
**Subtotal**	8/32 25%	16/32 52%
**Subtotal (normalized)**	9/35 26%	17/35 48%
**BMAT score (Sect. 1, norm.)**	**2.6 (≈ 15th perc.)**	**4.6 (≈ 74th perc.)**
**UCL applicants average score**	4.5	4.5
**UCL interviewed average score**	5.1	5.1
**UCL offer holders average score**	5.1	5.1
**Section 2: Scientific knowledge and applications**		
	**BMAT Oct. 2018 ChatGPT-3.5**	**BMAT Oct. 2018 ChatGPT-4**
**administered**	23/27	23/27
**Biology**	0/6 0%	3/6 50%
**Chemistry**	2/6 33%	2/6 33%
**Physics**	4/5 80%	4/5 80%
**Mathematics**	3/6 50%	2/6 33%
**Subtotal**	9/23 39%	11/23 48%
**Subtotal (normalized)**	11/27 41%	13/27 48%
**BMAT score (Sect. 2, norm.)**	**4.0 (≈ 51st perc.)**	**4.4 (≈ 64th perc.)**
**UCL applicants mean score**	4.6	4.6
**UCL interviewed mean score**	5.3	5.3
**UCL offer holders mean score**	5.3	5.3
**Total**	**17/55 31%**	**27/55 49%**

**Table 7:** BMAT results (November 2020). The table shows the results obtained by ChatGPT-3.5 (left) and ChatGPT-4 (right) in the November 2020 BMAT test. For Sections 1 and 2 of the test, we report the number of questions administered, the raw scores (correct answers/questions administered) in each subject area and in the whole section, the normalized raw score, and the corresponding BMAT score followed by the relative percentile. At the bottom of each section, the UCL applicants, interviewed, and offer holders average scores are reported. The bottom row shows the ratio of the total number of correct answers to the total number of questions administered and the corresponding percentage.

**Section 1: Thinking skills**		
	**BMAT Nov. 2020 ChatGPT-3.5**	**BMAT Nov. 2020 ChatGPT-4**
**administered**	26/32	26/32
**Critical thinking**	7/16 44%	15/16 94%
**Problem solving**	6/10 60%	7/10 70%
**Subtotal**	13/26 50%	22/26 85%
**Subtotal (normalized)**	16/32 50%	27/32 84%
**BMAT score (Sect. 1, norm.)**	**4.1 (≈ 40th perc.)**	**6.5 (≈ 95th perc.)**
**UCL applicants average score**	4.3	4.3
**UCL interviewed average score**	5.2	5.2
**UCL offer holders average score**	5.2	5.2
**Section 2: Scientific knowledge and applications**		
	**BMAT Nov. 2020 ChatGPT-3.5**	**BMAT Nov. 2020 ChatGPT-4**
**administered**	20/27	20/27
**Biology**	2/6 33%	3/6 50%
**Chemistry**	4/5 80%	3/5 60%
**Physics**	2/6 33%	4/6 67%
**Mathematics**	2/3 67%	1/3 33%
**Subtotal**	10/20 50%	11/20 55%
**Subtotal (normalized)**	13/27 48%	15/27 55%
**BMAT score (Sect. 2, norm.)**	**5.1 (≈ 69th-74th perc.)**	**5.7 (≈ 84th perc.)**
**UCL applicants average score**	4.1	4.1
**UCL interviewed average score**	4.8	4.8
**UCL offer holders average score**	4.8	4.8
**Total**	**23/46 50%**	**33/46 72%**

**Table 8:** BMAT results (November 2021). The table shows the results obtained by ChatGPT-3.5 (left) and ChatGPT-4 (right) in the November 2021 BMAT test. For Sections 1 and 2 of the test, we report the number of questions administered, the raw scores (correct answers/questions administered) in each subject area and in the whole section, the normalized raw score, and the corresponding BMAT score followed by the relative percentile. At the bottom of each section, the UCL applicants, interviewed, and offer holders average scores are reported. The bottom row shows the ratio of the total number of correct answers to the total number of questions administered and the corresponding percentage.

**Section 1: Thinking skills**		
	**BMAT Nov. 2021 ChatGPT-3.5**	**BMAT Nov. 2021 ChatGPT-4**
**administered**	25/32	25/32
**Critical thinking**	12/16 75%	14/16 87%
**Problem solving**	4/9 44%	2/9 22%
**Subtotal**	16/25 64%	16/25 64%
**Subtotal (normalized)**	20/32 62%	20/32 62%
**BMAT score (Sect. 1, norm.)**	**4.6 (≈ 64th perc.)**	**4.6 (≈ 64th perc.)**
**UCL applicants average score**	4.8	4.8
**UCL interviewed average score**	5.7	5.7
**UCL offer holders average score**	5.7	5.7
**Section 2: Scientific knowledge and applications**		
	**BMAT Nov. 2021 ChatGPT-3.5**	**BMAT Nov. 2021 ChatGPT-4**
**administered**	22/27	22/27
**Biology**	0/5 0%	4/5 80%
**Chemistry**	2/6 33%	3/6 50%
**Physics**	3/7 43%	3/7 43%
**Mathematics**	2/4 50%	1/4 25%
**Subtotal**	7/22 32%	11/22 50%
**Subtotal (normalized)**	9/27 33%	13/27 48%
**BMAT score (Sect. 2, norm.)**	**3.9 (≈ 45th-50th perc.)**	**4.9 (≈ 79th-89th perc.)**
**UCL applicants average score**	4.8	4.8
**UCL interviewed average score**	5.8	5.8
**UCL offer holders average score**	5.8	5.8
**Total**	**23/47 49%**	**27/47 57%**

Table 9 shows some aggregate percentages (specifically, the percentages of correct answers relative to the total number of questions submitted in each subject area in Sections 1 and 2 of the three tests, and the total aggregate percentages for these two sections), the average scores for Sections 1 and 2, and the average total score.

**Table 9:** BMAT results. Aggregate percentages and average scores for the 2018, 2020 and 2021 tests. Given the different structure of the 2018 test compared to the other two tests (see Section 2.2, point (1)), the “Understanding argument” area of the 2018 test has been considered equivalent to the “Critical thinking” area of the 2020 and 2021 tests, while the “Problem solving” and “Data analysis” areas of the 2018 test have been considered as a single area, which in turn has been taken to be equivalent to the “Problem solving” area of the 2020 and 2021 tests. For each subject area, the aggregate percentage is the ratio of correct answers to the total number of questions submitted in the three tests for that subject area. The total aggregate percentage on the bottom row is the ratio of correct answers to the total number of questions submitted in the three tests. Each section score is the average normalized section score for the 2018, 2020 and 2021 tests.

BMAT	Aggregate 2018, 2020, 2021	
	ChatGPT-3.5	ChatGPT-4
**Section 1: Aptitude and skills—Thinking skills**		
**Understanding argument—Critical thinking**	59%	88%
**Problem solving—Data analysis**	29%	41%
**Subtotal**	45%	65%
**BMAT score (Sect. 1, norm.)**	**3.9**	**5.2**
**Section 2: Scientific knowledge and applications**		
**Biology**	12%	59%
**Chemistry**	47%	47%
**Physics**	50%	61%
**Mathematics**	54%	31%
**Subtotal**	40%	51%
**BMAT score (Sect. 2, norm.)**	**4.4**	**5.0**
**Total**	**43%**	**59%**

In the 2018 test (Table 6), ChatGPT-3.5 scored 2.6 in Section 1 (≈ 15^*th*^ percentile and 1.9 below the average score of UCL applicants), and 4.0 in Section 2 (≈ 51^*st*^ percentile and 0.6 below the average score of UCL applicants); ChatGPT-4 scored 4.6 in Section 1 (≈ 74^*th*^ percentile and 0.1 above the average score of UCL applicants, but 0.5 below the average score of either UCL interviewed applicants or offer holders), and 4.4 in Section 3 (≈ 64^*th*^ percentile and 0.2 below the average score of UCL applicants).

In the 2020 test (Table 7), ChatGPT-3.5 scored 4.1 in Section 1 (≈ 40^*th*^ percentile and 0.2 below the average score of UCL applicants), and 5.1 in Section 2 (≈ 69^*th*^ percentile and 0.3 above the average score of either UCL intervied applicants or offer holders); ChatGPT-4 scored 6.5 in Section 1 (≈ 95^*th*^ percentile and 1.3 above the average score of either UCL interviewed applicants or offer holders), and 5.7 in Section 2 (≈ 81^*st*^ percentile and 0.9 above the average score of either UCL interviewed applicants or offer holders).

In the 2021 test (Table 8), ChatGPT-3.5 scored 4.6 in Section 1 (≈ 64^*th*^ percentile and 0.2 below the average score of UCL applicants), and 3.9 in Section 2 (≈ 35^*th*^ percentile and 0.9 below the average score of UCL applicants); ChatGPT-4 scored 4.6 in Section 1 (same score as ChatGPT-3.5), and 4.9 in Section 2 (≈ 64^*th*^−80^*th*^ percentile and 0.1 above the average score of UCL applicants, but 0.9 below the average score of either UCL intervied applicants or offer holders).

The results for each year show an improvement of ChatGPT-4 over ChatGPT-3.5 in both test sections, except Section 1 of the 2021 test. Moreover, it is worth noting that the aggregate results of ChatGPT-4 in the “Mathematics” subject area are worse that those of ChatGPT-3.5 (31% of correct answers vs. 54%, see Table 9).

Compared to the results on the SAT tests, the results here are more mixed, being excellent only for the 2020 test. In addition, the differences in performance between questions requiring mainly linguistic skills and those requiring scientific or logico-mathematical reasoning are confirmed. In particular, the aggregate results obtained in the “Understanding Argument-Critical Thinking” subject area are higher than those obtained in all the other subject areas (see Table 9).

### 6.3 IMSAT

Tables 10–12 show the results of administering the three IMSAT tests for the years 2019, 2020 and 2022 to the two versions of ChatGPT.

**Table 10:** IMSAT results 2019.

	IMSAT 2019 ChatGPT-3.5	IMSAT 2019 ChatGPT-4
**administered**	60/60	60/60
**General knowledge**	12/13 **92%**	13/13 **100%**
**Logical reasoning**	3/9 **33%**	1/9 **11%**
**Biology**	14/18 **78%**	17/18 **94%**
**Chemistry**	7/12 **58%**	5/12 **42%**
**Physics and Math**	3/8 **37%**	5/8 **62%**
**total**	39/60 **65%**	41/60 **68%**
**IMSAT score**	**50,1**	**53,9**
**bottom ranked school cutoff**	41,4	41,4
**top ranked school cutoff**	56,1	56,1
**admitted to school ranked**	**#4 Bologna**	**#2 Pavia**

**Table 11:** IMSAT results 2020. For this test we administered 59 questions instead of 60, because one of the biology questions was based on an image that could not be processed by either version of ChatGPT (see Section 4).

	IMSAT 2020 ChatGPT-3.5	IMSAT 2020 ChatGPT-4
**administered**	59/60	59/60
**General knowledge**	11/13 **85%**	13/13 **100%**
**Logical reasoning**	2/9 **22%**	3/9 **33%**
**Biology**	16/17 **94%**	16/17 **94%**
**Chemistry**	6/12 **50%**	9/12 **75%**
**Physics and Math**	4/8 **50%**	5/8 **62%**
**total**	39/59 **66%**	46/59 **78%**
**IMSAT score**	**50,5**	**63,8**
**bottom ranked school cutoff**	39,5	39,5
**top ranked school cutoff**	57,8	57,8
**admitted to school ranked**	**#5 Padova**	**#1 Bicocca**

**Table 12:** IMSAT results 2022.

	IMSAT 2022 ChatGPT-3.5	IMSAT 2022 ChatGPT-4
**administered**	60/60	60/60
**General knowledge**	4/4 **100%**	4/4 **100%**
**Logical reasoning**	1/5 **20%**	3/5 **60%**
**Biology**	16/23 **70%**	20/23 **87%**
**Chemistry**	9/15 **60%**	11/15 **73%**
**Physics and Math**	6/13 **46%**	3/13 **23%**
**total**	36/60 **62%**	41/60 **68%**
**IMSAT score**	**44,4**	**53,9**
**bottom ranked school cutoff**	33,4	33,4
**top ranked school cutoff**	54,5	54,5
**admitted to school ranked**	**#8 Bologna-Forlì**	**#2 Bologna**

We see that, for each year (Tables 10–12), the score of ChatGPT-4 is higher than that of ChatGPT-3.5, even if, for some subject areas, there is no improvement, or even a decline, on some of the three tests.

In addition, it is very clear (see Table 13) that the overall performance of both versions of the chat drops significantly when it comes to questions of logic, but also of physics and mathematics. Even if ChatGPT-4 improves its performance in these areas compared to ChatGPT-3.5, the aggregate results of the three tests are still quite low.

**Table 13:** IMSAT results. Aggregate percentages of correct answers for the 2019, 2020 and 2022 tests. For each subject area and each chat model, the aggregate percentage is the ratio of the number of correct answers to the total number of questions administered in the three tests in that subject area. The total aggregate percentage on the bottom row is the ratio of correct answers to the total number of questions administered in the three tests.

	IMSAT Aggregate 2019, 2020, 2021	
	ChatGPT-3.5	ChatGPT-4
**General knowledge**	90%	100%
**Logical reasoning**	26%	30%
**Biology**	79%	91%
**Chemistry**	56%	64%
**Physics and Math**	44%	44%
**Total**	64%	72%

For each of Tables 10–12, the last row shows the highest ranked medical school to which Chat-GPT’s score would have granted admission, according to the national ranking for the specific year. The two upper rows show the cutoff scores of the lowest and highest ranked university, respectively.

Interestingly, in every year, the test score of either ChatGPT-4 or ChatGPT-3.5 would have given admission to the top eight Italian medical schools (out of a total of about 43. The total number of medical schools in the national ranking of the Ministry of University and Research varies from year to year: 38 in 2019, 43 in 2020, 49 in 2022).

However, the admission record of ChatGPT-4 is much better than that of ChatGPT- 3.5. In particular, they would have been admitted to the 2nd and 4th medical schools respectively in 2019, to the 1st and 5th in 2020, and to the 2nd and 8th in 2022.

## 7 Discussion

In the first part of this section, we discuss the results of the experiments described in Sections 3–5. In the second part, we offer a a classification and interpretation of common errors that emerged from the experiments, through a qualitative analysis of incorrect answers that reveal peculiar difficulties.

### 7.1 Performance evaluation

The aggregate results of our experiment (Tables 2, 9, 13) show that both versions of ChatGPT had the best overall performance on the three SAT tests (72% of correct answers for ChatGPT-3.5, 86% for ChatGPT-4), followed by the IMSAT tests (64%, 72%), and then by the BMAT tests (43%, 59%). They also show a clear improvement of ChatGPT-4’s overall performance over ChatGPT-3.5’s in all tests (increase ratios: SAT +19%, IMSAT +13%, BMAT +37%).

In the IMSAT tests (Table 13), both versions of ChatGPT had the best performance in the General knowledge area (ChatGPT-3.5 90%, ChatGPT-4 100%), the worst in Logical Reasoning (26%, 30%). The two versions had the same order of performance also in the three subject areas that are aimed at testing knowledge and skills in science disciplines. The best performance for both versions was in Biology (ChatGPT-3.5 79%, ChatGPT-4 91%), followed by Chemistry (56%, 64%) and then Physics and Mathematics (44%, 44%).

In the BMAT tests (Table 9), for both versions of ChatGpt, the best performance was in Understanding argument-Critical thinking (ChatGPT-3.5 59%, ChatGPT-4 88%); the worst in Biology (12%) for ChatGPT-3.5, and in Mathematics (31%) for ChatGPT-4. However the second worst performance for both versions was in Problem Solving-Data analysis (ChatGPT-3.5 29%, ChatGPT-4 41%). In the other subject areas, in descending order, performance was as follows ChatGPT-3.5: Mathematics (54%), Physics (50%), Chemistry (47%); ChatGPT-4: Physics (61%), Biology (59%), Chemistry (47%).

In the SAT tests (Table 2), for both versions of ChatGpt, the best performance was in the Reading part (ChatGPT-3.5 78%, ChatGPT-4 95%); the worst in the Math part with calculator (63%, 75%). The two versions had the same order of performance also in the other two parts. The second best performance for both versions was in Writing (ChatGPT-3.5 73%, ChatGPT-4 87%), the second worst in Math no calculator (68%, 80%).

We observe that the type of knowledge and skills required by the parts of the three tests where both versions of ChatGPT performed best (IMSAT General knowledge, BMAT Understanding argument-Critical thinking, SAT Reading and Writing) are of roughly the same type. In fact, they are largely related to understanding language and using it appropriately in non-specialized contexts.

Conversely, if we look at the parts of the three tests where the two versions of ChatGPT had the worst performance, we see that they all require either some kind of specialized knowledge (BMAT: worst performance in Biology for ChatGPT-3.5, in Mathematics for ChatGPT-4.0), or some level of specialized symbolic skills, namely logical skills (IMSAT: worst performance in Logical reasoning for both ChatGPT versions), algorithmic skills (SAT: worst performance in Math with Calculator for both ChatGPT versions), or problem solving skills (BMAT: second worst performance in Problem solving-Data analysis for both ChatGPT versions).

These observations are consistent with a qualitative analysis that we made of a number of answers produced by the two versions of ChatGPT in the three tests (see Section 7.2 and the *Note on Remarkable Errors* in S1 File, p. 990). Thus, ChatGPT performs best when it comes to using primarily linguistic skills and knowledge, or even specialized knowledge that does not require particulary sophisticated skills to be applied appropriately to the question posed. Conversely, the greatest difficulties arise when the correct answer can only be produced by a non-obvious reworking of previously acquired knowledge, using symbolic skills (e.g., logical, algorithmic, specific problem solving skills, etc.) that are not purely linguistic but belong to a specific domain. This is consistent with the results obtained in other similar studies [4].

This observation could also explain the apparent differences found in the results obtained by both versions of ChatGPT on corresponding parts of the IMSAT and the BMAT tests. For example, the large discrepancy between the aggregate results in the respective Biology parts of the two tests (ChatGPT-3.5: 79% IMSAT, 12% BMAT; ChatGPT-4: 91% IMSAT, 59% BMAT) could be explained by two factors. First, the prevalence of questions in the IMSAT Biology part that are essentially notional in nature, and second, the fact that most of the questions in the BMAT Biology part seem to require a higher level of elaboration for an adequate answer to be produced.

Given these results, ChatGPT should show significant weaknesses when tested on symbolic skills of specific types. We got a confirmation of this prediction by testing ChatGPT’s ability to multiply pairs of natural numbers with an increasing number of digits. In Experiment 1 we proposed to ChatGPT-3.5 and to ChatGPT-4 ten sets of multiplications each consisting of fifty randomly chosen pairs of factors, the first with three digits and the second with two digits. Then, in Experiment 2, we increased the procedural complexity by administering to both models ten multiplication sets each consisting of fifty randomly chosen pairs of three-digit factors. The results are shown in Table 14.

**Table 14:** Average percentages of correct answers and standard deviations in ten repetitions of Experiments 1 and 2. (For further data and graphs see the *Note on Remarkable Errors in* S1 File, p. 990.).

	Exp. 1 avg	Exp. 2 avg
**ChatGPT-3.5**	74.4 +/- 8.04%	52.8 +/- 30.22%
**ChatGPT-4**	87 +/- 5.16%	36.6 +/- 4.82%

In Experiment 1, ChatGPT-4 performances are slightly better than ChatGPT-3.5 and the difference between the respective standard deviations is quite small. In Experiment 2, ChatGPT-3.5 seems to outperform ChatGPT-4 but the difference between the respective standard deviations is much greater. However, as the error intervals (2 standard deviations each) of both experiments overlap, the differences are not statistically significant.

These results confirm the hypothesis about the specific weaknesses of ChatGPT in solving problems of high computational complexity. In fact, for ChatGPT-4, a significant performance degradation due to the increase in computational complexity is observed between Experiment 1 and 2. For ChatGPT-3.5, the performance difference between the two experiments seems to be much smaller, but it is not statistically significant.

These results are also consistent with the worst performance of both versions of ChatGPT on the SAT tests being in the calculator math part, as noted above.

We like to remark how our analysis provides a detailed and comprehensive framework that is currently absent in the existing literature. We emphasize that the data discussed herein represent, for the first time, a fine-grained analysis of ChatGPT’s performance on tests commonly administered to medical school students. Specifically, the novelty of our methodology lies in: i) considering both aggregated and specific data, categorized by each subfields of topics; ii) comparing ChatGPT’s performance with the average results obtained by real students; iii) performing a detailed comparison between ChatGPT-3.5 and ChatGPT-4, highlighting strengths and weaknesses. This results in a detailed and comprehensive comparative framework that offers certainly innovative information compared to previous literature.

### 7.2 Qualitative analysis

In this section, we will take a closer look at the recurrent errors made by ChatGPT in the experiments conducted, in order to identify a set of error types and thus provide an overview of the model’s weaknesses. We also argue that the classification of recurrent errors is a useful contribution to existing pedagogical research on the use of this type of AI systems in education. We believe that the awareness of possible errors, as well as the ability to structure problems that take into account specific constraints, allows for a better use of this type of AI systems and may enhance their effective use in education.

#### 7.2.1 Frequent types of errors

In section 5 we have shown that ChatGPT performs quite well in the university admission tests considered. By comparing ChatGPT-3.5 and ChatGPT-4, it was possible to highlight the question types where the latter is superior to the former. However, it was also possible to identify some weaknesses in the more advanced version, whose performance on certain types of questions did not improve significantly compared to the previous version. Given these weaknesses of ChatGPT-4, we evaluated the possibility of describing and categorizing the problems found in the behavior of the model.

Identifying and classifying the type of errors most frequently made by the model is important for several reasons. First, from a technical point of view, it allows us to identify the weaknesses of the model in order to overcome them. Second, from a user’s point of view, it can help to create prompts that are easier for the model to handle. Third, from a pedagogical point of view, it can be used to formulate AI-proof tasks or, more generally, to maintain robust evaluation processes.

We have identified some recurrent types of errors made by ChatGPT-4 (hereafter ChatGPT) during the experiment reported in Section 4. In particular, we isolated a number of errors of a logical nature that affect the reliability of the model, which we categorize into three types: (1) “dogmatic” choice of solution procedure; (2) natural language formalization; (3) processing order:

1. ChatGPT never shies away from trying to give an answer, so it is not possible to estimate the degree of correctness the model entrusts to its own answer. It seems that ChatGPT relies on a specific procedure and lacks a wider range of plans. This makes it impossible to determine whether the chosen procedure is adequate or whether the data and tools at its disposal are insufficient to answer the question. This type of error is generally common to all incorrect answers. For some types of answers, this problem is highlighted by the fact that ChatGPT arrives at a result that is not on the list of possible alternatives. Very often, when ChatGPT finds a result that is not among the possible alternatives and is therefore obviously wrong, it does not try to use a different procedure, but simply chooses the answer that is closest to the one it found (as shown in example 1, 4 and 6 below). However, it is important to note that recent changes to the model now allow ChatGPT to offer more than one answer to a given question, and for the user to provide feedback on the choices they consider most appropriate, although it is not clear in which cases this feature is made available and why.
2. ChatGPT is sometimes unable to provide an appropriate truth table for simple formalized propositions and to correctly deduce conclusions from clear and explicit premises. Moreover, it often fails to evaluate an argument from a purely syntactic perspective if it is not explicitly asked to do so. Finally, when a question requires it to represent an intersection of given properties, it often fails to give the correct answer (see examples 2, 3 and 6).
3. ChatGPT encounters considerable difficulties with the notion of order in several cases. In problems involving an ordinal data distribution, the exact distances between positions in the order are sometimes not recognized and, consequently, the chosen procedure turns out to be incorrect. More specifically, these difficulties involve interpreting a position in the sequence in a way that is consistent with the available data, recognizing correct distances between positions in the sequence, following a given order according to the steps indicated, connecting the results of different calculations, and correctly tracing the steps of the process in order to match the results with the corresponding requirements. More generally, it has difficulty when asked to compute sequences of operations involving many procedural steps (see Examples 1, 3, 4 and 5).

In the following, we show and comment on some examples in which the types of errors described above are made visible. These examples are particularly significant because each of them contains one or more of the errors we have identified. Furthermore, despite their apparent simplicity, they show that these weaknesses are extremely sensitive to the structural complexity of the question presented, since even small increases in complexity can affect the correctness of the answer. Some of the examples are taken from the IMSAT test. We translated the Italian questions to which ChatGPT gave incorrect answers into English. Since the English answers to the translated questions were also incorrect and, modulo the translation, identical to the Italian ones, language competence did not appear to be a factor in determining the errors considered in our analysis.

- EXAMPLE 1 (BMAT 2018, Sect. 1 Q. 1, correct answer: B)
**Question**
  Riverside Walk is a popular walking path, 3.2 kilometers long, beside the River Bee. There is a seat situated at each end of the path and also every 400 meters in between. There are two litter bins just next to every seat and one litter bin every 100 meters between each seat and the next. How many litter bins are there along Riverside Walk?
  A) 40
  B) 42
  C) 45
  D) 48
  E) 51
**Answer**
  To determine the number of litter bins along Riverside Walk first calculate the numbers of seats. There are seats and both ends and every 400 meters in between: 3,2 km/0,4 KM = 8 intervals There are 8 intervals, so there 9 seats (one at each end and one at each 7 intervals) There are two litter bins next to every seat: 9 seats* 2 litter bins = 18 litter beans. There is one litter bin every 100 meters between each seat and the next. There are 8 intervals between the seta and 4 litter bins per interval: 8 intervals*4 litter binds = 32 litter bins.
  Adding the litter bins by the seats and those between the seats:
  18 + 32 = 50 litter bins
  The answer closest to 50 is E 51.
**Comment**
  This example shows that ChatGPT is not able to fulfill the given conditions and settles for an approximate answer (a case of type 3 error).

- EXAMPLE 2 (BMAT 2021, Sect. 1, Q. 15, correct answer: E)
**Question**
  Any hospital which is serious about reducing its deficit and bringing its budget under control will examine carefully the land and buildings it owns, in order to see if any of them can be sold to raise money. Wildflower General Hospital is evidently determined to keep its finances under control, because it has recently cancelled a planned extension to the visitors’ car park and sold the ground for housing. Which one of the following most closely parallels the reasoning used in the above argument?
  A) Any student who wants to gain a place at a top university should develop a special interest which will impress admissions tutors. Emily aims to secure a place at a top university. So she needs to develop an impressive special interest.
  B) Any parent who wishes to bring up children to be truthful should never lie to them. Natalie cannot want her children to grow up to be truthful, because she often deliberately tells them things which are not true.
  C) Any school which persistently gets poor examination results will be put into ‘special measures’ by the Government. Edgewater Secondary School is sure to be put into special measures because its exam results have been dreadful for the last 5 years.
  D) Any worker at this company who is persistently late for work will be dismissed. Joe always arrives at work on time. So he is in no danger of losing his job.
  E) Anyone who wishes to study English at university needs to learn to read quickly. Vanessa has developed impressive speed-reading skills. So she must be planning to study English at university.
**Answer**
  The correct answer is B. The reasoning in the original argument is that if a hospital is serious about reducing its deficits it will examine its assets and consider selling them. The argument the n states that Wildflower General Hospital is doing this so it must be serious about reducing its deficit. Answer B follows the same reasoning: if a parent wants to raise truthful children they should never lie to them. The argument then states that Natalie often lies to her children, so she must want them to be truthful.
**Comment**
  In this example ChatGPT fails to analyze arguments from a formal point of view. Instead of analyzing the logical structure of the argument, it looks for analogies between the meanings of the words involved, showing a kind of “semantic bias” (error type 2).

- EXAMPLE 3 (IMSAT 2020, Q. 7, correct answer: D)
**Question**
  How many numbers between 1 and 8, inclusive, have at least two of the following three attributes: 1 being odd, 2 being greater than or equal to 6, 3 and being less than or equal to 6?
  A) 2
  B) 6
  C) 4
  D) 5
  E) 3
**Answer**
  Let’s examine the properties that we must consider: 1 being odd, 2 being greater than or equal to 6, 3 and being less than or equal to 6.
  Now, Let’s examine numbers from one to eight and their properties:
  1 only the property 1
  2 no properties
  3 only the property 1
  4 no properties
  6 property 2 and 3
  7 the property 1 and 2
  8 no properties
  Numbers between 1 and 8, inclusive, which possess at least two of the properties are 5,6,7, so correct answer is E)3.
**Comment**
  This example shows that ChatGPT struggles to compute even very simple problems that require the representation of set intersections and the evaluation of elements that verify structured compositions of properties. In this case, it fails to recognize that the numbers 1 and 3 correspond to two of the specified properties (a case of error type 2).

- EXAMPLE 4 (BMAT 2018, Sect. 1, Q. 7, correct answer: D)
**Question**
  The lift in the hotel I am staying in takes 3 seconds to move between consecutive floors and when it stops at a floor it remains stationary for a minimum of 9 seconds to let people in and out. A screen inside the lift shows a list of the floors that it has been requested to stop at. When the lift moves off after stopping at a floor it always stops next at the closest floor on its list. If two floors are equally close in both directions, it continues in the direction in which it was previously travelling. The door of the lift has just closed after I have entered it at floor 11 and added floor 4 to the list of floors to be stopped at. The other floors currently on the list are floors 1, 6, 15 and 24. Assuming that no further floors are added to the list, what is the minimum time that will elapse before the lift door opens at floor 4?
  A) 63 seconds
  B) 90 seconds
  C) 99 seconds
  D) 126 seconds
  E) 153 seconds
**Answer**
  To calculate the time it takes for the lift to reach floor 4 we need to determine the order of floors it will stop at; given the conditions stated. The current list on the floor is 1, 4, 6, 15 and 24. The lift start and floor 11 first stop: the closest floor to the floor 11 is floor 6
  Time to floor 6: 3 sec/floor*(11 -6) floors = 15 seconds (travel time) + 9 seconds
  (stationary time) = 24 seconds
  Second stop: the closest floor to the floor 6 is floor 4
  Time to floor 4: 3/seconds/floor*(6—4) floors = 6 seconds (travel time) + 9 seconds
  (stationary time) = 15 seconds
  Add the time spent to reach each floor:
  24 seconds (to floor 6) + 15 seconds (to floor 4) = 39 seconds
  The minimum time that will elapse before the lift opens at floor 4 is A) 63 seconds.
**Comment**
  This example shows that ChatGPT has trouble correctly representing orders of data, their distance in a series, or identifying an exact position within sequences and its distance from other positions. Specifically, ChatGPT does not recognize that the 15th floor is closer to the current floor (11th) than the 6th floor (a case of error type 3). Also, the result of its calculation (73 seconds in total) does not match any of the given answers, but it still chooses one from the list (error type 1).

- EXAMPLE 5 (IMSAT 2020, Q. 18, correct answer: A)
**Question**
  Enea and his friends, less than ten people in total, go to a restaurant with a fixed-price menu: the price for a meat main course is €11 and for a fish main course €13. If Enea and his friends spent €107 on main courses, how many fish main courses did they order?
  A) 4
  B) 5
  C) 2
  D) 9
  E) 7
**Answer**
  Let X be the meat and Y the fish main courses ordered by Aeneas and his friends. We know that a main course of meat costs €11 and a main course of fish costs €13. We also know that Aeneas and his friends spent a total of 107 € on the main courses. We can write an equation for the total cost of the main courses:
  11 x + 13 y = 107
  We also know that the total number of people is less than 10, so the sum of X and Y is less than 10.
  X+Y < 10
  We now need to find a combination of X and Y that satisfies both equations; we will try different solutions for X and Y, bearing in mind that they must be positive integers.
  1 X = 1, Y = 8: 11(1)+13(8) = 115 (it doesn’t satisfy cost equation)
  2 X = 2, Y = 7: 11(2)+13(7) = 107 (it satisfies cost equation)
  3 X = 3, Y = 6: 11(3)+13(6) = 99 (it doesn’t satisfy cost equation)
  4 X = 4, Y = 5: 11(4)+13(5) = 115 (it doesn’t satisfy cost equation)
  The only combination which satisfies both equations is: X = 2, Y = 7. Aeneas and his friends ordered 7 fish main courses
  Correct answer is E)7
**Comment**
  In this example, ChatGPT sets up the problem correctly, but then it does not continue the calculation until the end of the process, considering only some of the possible cases. Moreover, it does not realize that the given answer is wrong (in fact, (11×2) + (13×7) = 113, not 107). This can be seen as a case of problems caused by procedural complexity (error type 3).

- EXAMPLE 6 (BMAT 2018, Sect. 1, Q. 23, correct answer: B)
**Question**
  I use a 4-digit PIN with my bank cards. Each digit can be any digit between 1 and 9. I have set the code so that:
  - the first and second digits are consecutive (subtracting the first from the second digit gives 1).
  - the third and fourth digits are consecutive (subtracting the third from the fourth digit gives 1).
  - the first and fourth digits add together to give the same value as the second and third digits multiplied together.
  What is the last (fourth) digit of my PIN?
  A) 2
  B) 3
  C) 4
  D) 5
  E) 6
  F) 7
  G) 8
  H) 9
**Answer**
  Let’s represent the Pin by four variables: A, B, C and D, where A is the first digit, B is the second digit, C is the third digit, and D is the fourth digit. The given conditions are as follows:
  1. B = A + 1
  2. D = C + 1
  3. A + D = B * C
  Now, substitute condition 1) and 2) into condition 3 to get:
  A + (C+1) = (A+1) * C
  A + C + 1 = AC + C
  A − AC = -C (Move AC and C to the left-hand side)
  Now, factor out A and C:
  A(1-C) = -C
  Since A and C are digits between 1 and 9, and A = C − 1, the possible pairs are:
  A, C = 1, 2
  A, C = 2, 3
  A, C = 3, 4
  A, C = 4, 5
  A, C = 5, 6
  A, C = 6, 7
  A, C = 7, 8
  A, C = 8, 9
  Now, find the pairs that satisfy A(1-C) = C:
  1(1–2) = -2 (satisfies)
  2(1–3) = -3 (satisfies)
  3(1–4) = -4 (satisfies)
  4(1–5) = -5 (satisfies)
  5(1–6) = -6 (satisfies)
  6(1–7) = -7 (satisfies)
  7(1–8) = -8 (satisfies)
  8(1–9) = -9 (satisfies)
  All pairs satisfy the condition, so D can take values 3, 4, 5, 6, 7, 8, or 9. The last digit of the PIN is D. The answer is H 9.
**Comment**
  In this example, ChatGPT’s response clearly reveals two of the types of errors we identified. First, we notice two errors related to computational complexity (error type 2): 1) With the complex procedure used, we almost immediately lose sight of the fact that the number to be identified is the last one. In fact, the equation *A*(1 − *C*) = −*C* can in no way be used to derive the value of the last number of the PIN code, i.e. D, in the notation used by ChatGPT. 2) The results of all operations performed to derive the pairs satisfying the equation *A*(1 − *C*) = −*C* are incorrect.
  Finally, despite the fact that the procedure does not lead to a unique result, instead of changing it, ChatGPT does not hesitate to give an answer, never questioning the procedure used (a clear example of a type 1 error).

#### 7.2.2 Potential implications for education

The previous results may help to identify the strategies that can best exploit the potential of ChatGPT. Although the aim of our research is not to provide a set of rules for the use of these technologies in education, we believe that the analysis of ChatGPT’s strengths and weaknesses is a necessary step in view of its use in educational contexts.

To date, ChatGPT has been used in various contexts related to education, for example, to support learning, to generate complex and deep answers for exams, to translate and paraphrase texts, etc. For a review of these applications, see [19]. In a meta-analysis of the impact of AI chatbots on student learning outcomes, [20] highlights that the use of these technologies has a greater improvement effect on students in higher education compared to those in primary education. In addition, it was found that the learning curve rises abruptly in the early stages of technology use, while it tapers off with longer interventions, a phenomenon explained by a novelty effect.

A systematic review of the literature on the impact of the use of ChatGPT in education [21] shows the positive impact of this technology on the teacher learning process and highlights the importance of training teachers to use this kind of AI systems. In our opinion, this should be better clarified, because what should be sought is not only the correct use of the system in general, but also its correct use as a teaching tool. To this end, some studies point to the need for a serious analysis of the relationship between human teachers and AI technologies [22] and propose a set of technical, cognitive and meta-cognitive strategies useful for the conscious implementation of these tools in education [23]. As we have shown, ChatGPT has very good linguistic skills, but many weaknesses in solving other kinds of problems. The awareness of these strengths and weaknesses will allow teachers to use ChatGPT more advertently. For example Therefore, a natural application area of the critical analysis offered in our study is the training of teacher educators in strategies that enable them to use chatGPT more effectively.

The literature also shows considerable, and in some ways excessive, enthusiasm for the educational use of this or similar AI systems, as if the introduction of such a powerful technology should in itself entail a complete overhaul of the techniques and tools used in educational science [24]. Instead, we believe that the focus at this stage of research should be on the critical and informed use of these technologies (see, for example, [25]), and to this end it is necessary to first identify clearly their strengths and weaknesses. We conclude this section by highlighting the importance and innovativeness of the approach used here. After conducting a specific quantitative analysis in Section 7.1, we identified and analyzed (in Section 7.2) three specific types of logical challenges that ChatGPT faces with difficulty. We believe this not only represents an innovative contribution to the interplay between logic and artificial language models but also has the potential to expand the debate to new and stimulating questions regarding the connection between language and artificial intelligence. Another innovative aspect of our work, which constitutes an advancement over the current state of the art, is the potential use of the results obtained in the educational context. Some innovative insights have been introduced in Section 7.2.1, which highlights both the legitimate aspirations of ChatGPT as an educational support tool and potential areas of concern. For this reason, we believe that this work, through a careful analysis of both quantitative and qualitative aspects, constitutes a significant advancement over the existing literature and paves the way for interesting research developments, as will be further detailed in the next section.

## 8 Conclusions and open issues

The analysis of the recurrent errors made by ChatGPT shows that, contrary to what is usually done in other areas of AI (such as automated theorem proving, chess analysis, etc.), it is not possible to rely on such models by simply taking the answers they provide at face value, but that some adequate training is required to use these technologies in our workflow. Specific skills and good competencies are required in the different application contexts where ChatGPT is used. On the one hand, good domain knowledge is required to judge the correctness of the answer given or the appropriateness of the procedure indicated to solve the problems presented. On the other hand, specific skills are required to identify the error, understand its cause, and try to implement a possible correction process, guided by the structuring of appropriate prompts. We believe that it is important to recognize the weaknesses and develop expertise in dealing with common and structural errors that ChatGPT makes when solving certain problems. This can be a useful element in approaching these language models with greater awareness and effectiveness.

In order to refine the behavior of ChatGPT and gradually overcome its current limitations, developers are currently pursuing several directions in parallel, depending on the specific use cases and application domains. Two key development directions are the integration of ChatGPT with Application Programming Interfaces (APIs) and the practice of prompt engineering.

The integration of ChatGPT with Application Programming Interfaces (APIs) is expected to revolutionize the landscape of human-computer interaction in various applications [26]. As natural language processing capabilities continue to improve, this synergistic relationship will enable more seamless and efficient communication between humans and machines, as well as across multiple digital platforms. The potential applications of this integration are vast, ranging from personalized virtual assistants and customer support to real-time language translation and intelligent content generation. In addition, the combination of ChatGPT and APIs will pave the way for new approaches to data analysis, fostering a new era of data-driven decision making. By facilitating machine-to-machine communication and automating routine tasks, this powerful alliance promises to catalyze scientific research, optimize business operations and improve the overall user experience in the digital realm, effectively revolutionizing much of the world of work.

On the other hand, prompt engineering has also emerged as a natural progression to optimize the performance and user experience of ChatGPT systems [27]. Prompt engineering is the process of refining input queries to elicit more accurate and relevant responses from AI models. This technique allows for better communication between humans and AI systems, ultimately resulting in a more satisfying user experience. By creating carefully structured prompts, users can guide the model toward the desired outcome, effectively tapping into the model’s vast knowledge. We find that logic can play a key role in prompt engineering, as it is essential for disambiguating queries and ensuring coherent responses. Integrating logic into the model could enable ChatGPT to

- understand and interpret context. By incorporating logic, the AI system can better recognize the context of a conversation, enabling more meaningful interactions and responses;
- handle complex queries. Logical reasoning gives ChatGPT the ability to handle more complex queries, such as those that require multi-step reasoning or involve multiple facts or arguments;
- handle contradictions. A strong foundation in logic enables AI models to identify and address inconsistencies or contradictions in the information provided, resulting in more accurate responses;
- improve user experience. By using logical reasoning, ChatGPT systems can better understand user intent, resulting in improved responses and overall user satisfaction.

Therefore, the integration of prompt engineering and logic may represent a natural evolution in the field of AI language models. By using these techniques, users can effectively leverage the vast knowledge of GPT models, resulting in more accurate, relevant and coherent responses. As ChatGPT systems continue to evolve, the importance of prompt engineering and logic will only increase, ultimately contributing to more refined and efficient AI-human communication. Logic can also play an essential role in multi-level prompt engineering, where the user interacts with the chat by providing a new prompt following the chat’s response based on a previous prompt, enabling a more effective dialogue between the user and machine.

We believe that it is important to recognize the weaknesses of the model and to develop expertise in addressing common and structural errors that ChatGPT makes in solving particular problems. This can be a useful element in approaching these language models with greater awareness and effectiveness, and may have important implications for the educational use of such AI systems. In this area, further research is needed to distinguish those areas where these systems can already be used for educational purposes (such as generating questions or topic suggestions for exams, using them as study aids, etc.) from those where their use in this regard is still doubtful. In addition, specific studies are needed to understand how to prepare teachers for the introduction of these tools in specific disciplines.

Our results highlight the fact that specific skills and competencies are needed on the part of the user in order to use ChatGPT effectively in a specific context. In particular, domain-specific skills are required to assess the correctness of the answers it provides or the appropriateness of the procedures it suggests for solving the problems posed. On the other hand, specific skills are required to identify an error, understand its nature and try to implement a possible correction process by structuring appropriate prompts.

The limitations of our work are mainly the following: (i) given the ongoing rapid progress of these technologies, our conclusions may be distorted in the near future by their improvements; (ii) at the current stage of research, we are unable to give exact prescriptions for the professional use of ChatGPT, but can only try to make suggestions based on the evaluation of some of its capabilities and the reliability of its answers in relation to the different types of questions in the university tests considered.

We are continuing to experiment with ChatGPT to obtain better quantitative and qualitative data on its performance and specific strengths or weaknesses. We also believe that this type of research raises additional and pressing questions that deserve to be discussed and explored in a broader context. One of the most pressing questions concerns the kind of skills and knowledge that it is appropriate today to require of a student about to enter university level studies, in a context in which interaction with intelligent systems of the ChatGPT type will become increasingly common and inevitable.

## Supporting information

S1 File All experiments conducted on ChatGPT-3.5 and ChatGPT-4, which are the subject of this study, are included in this file. (PDF)
